# Selection of risk assessment methods for osteoporosis screening in postmenopausal women with low-energy fractures: A comparison of fracture risk assessment tool, digital X-ray radiogrammetry, and dual-energy X-ray absorptiometry

**DOI:** 10.1177/20503121211073421

**Published:** 2022-01-15

**Authors:** Mischa Woisetschläger, Simona Chisalita, Marta Vergara, Anna Spångeus

**Affiliations:** 1 Department of Radiology in Linköping, and Department of Health, Medicine and Caring Sciences, Linköping University, Linköping, Sweden; 2 Center for Medical Image Science and Visualization (CMIV), Linköping University, Linköping, Sweden; 3 Department of Endocrinology in Linköping, and Department of Health, Medicine and Caring Sciences, Linköping University, Linköping, Sweden; 4 Department of Acute Internal Medicine and Geriatrics in Linköping, and Department of Health, Medicine and Caring Sciences, Linköping University, Linköping, Sweden

**Keywords:** Osteoporosis, bone mineral density, DXA, FRAX, vertebral fractures, diagnostic methods, musculoskeletal conditions

## Abstract

**Objectives:**

Fracture liaison services are designed to identify patients needing osteoporosis treatment after a fracture. Some fracture liaison service designs involve a prescreening step, for example, fracture risk assessment tool (FRAX^®^). Another possible prescreening tools are bone mass density assessment in the acute setting. The aim of this study was to assess the effectiveness of prescreening tools.

**Methods:**

In the present prospective cohort study, women aged >55 years with a radius fracture were included. Patients were recruited at the emergency department after experiencing their fracture. All patients performed fracture risk assessment by fracture risk assessment tool, and bone mass density assessment by digital X-ray radiogrammetry and dual-energy X-ray absorptiometry (prescreening steps) as well as full routine evaluation at the osteoporosis unit (endpoint). The main outcome measures were sensitivity, specificity, predictive values, and area under the curve.

**Results:**

Forty-one women were recruited (mean age: 70 ± 8 years). Of these, 54% fulfilled the treatment indication criteria of osteoporosis after a full examination. Fracture risk assessment tool without bone mass density (cutoff ⩾ 15%) for prescreening patients had a high sensitivity (90%) but a low area under the curve (0.50) and specificity (16%). The highest area under the curve (0.73) was found prescreening with bone mass density assessment (dual-energy X-ray absorptiometry or digital X-ray radiogrammetry) having a sensitivity of 59%–86% and specificity of 61%–90%.

**Conclusion:**

This study, though small, raises questions regarding the effectiveness of using a prescreening step in fracture liaison services for high-risk individuals. In this cohort, FRAX^®^ without bone mass density had a low precision, with a risk of both underestimating and overestimating patients requiring treatment. Bone mass density assessment in the acute setting could improve the precision of prescreening. Further investigations on the effectiveness and health economics of prescreening steps in fracture liaison services are needed.

## Introduction

Osteoporosis and the subsequent increased risk of fragility fractures are common, with about 9 million fragility fractures occurring yearly worldwide.^1^ These fractures are associated with considerable morbidity and increased mortality^2–4^ as well as a high economic burden for society, with an estimated annual cost of about 37 billion Euros in the 27 EU countries.^5^ There are several cost-effective treatment options on the market.^6^ However, despite osteoporotic fractures being common and causing substantial morbidity and a high economic burden for society, osteoporosis is still a highly underdiagnosed and undertreated disease. In Sweden, only about 15% of women aged >50 years are medically treated for osteoporosis within 6–12 months of experiencing an osteoporotic fracture.^4^

National and international efforts are being made to increase this figure, for example, by using fracture liaison services (FLSs)^7–9^ to ensure adequate evaluation and osteoporosis treatment initiation after a fracture. Some FLSs involve prescreening steps (involving a clinical risk factor (CRF) assessment) to decide which patients should be offered a full examination (including dual-energy X-ray absorptiometry (DXA), vertebral fracture assessment (VFA), and CRF assessment) and which patients should not be assessed (i.e. which patients should be declared healthy/at low risk of further fractures).

**Figure 1.**
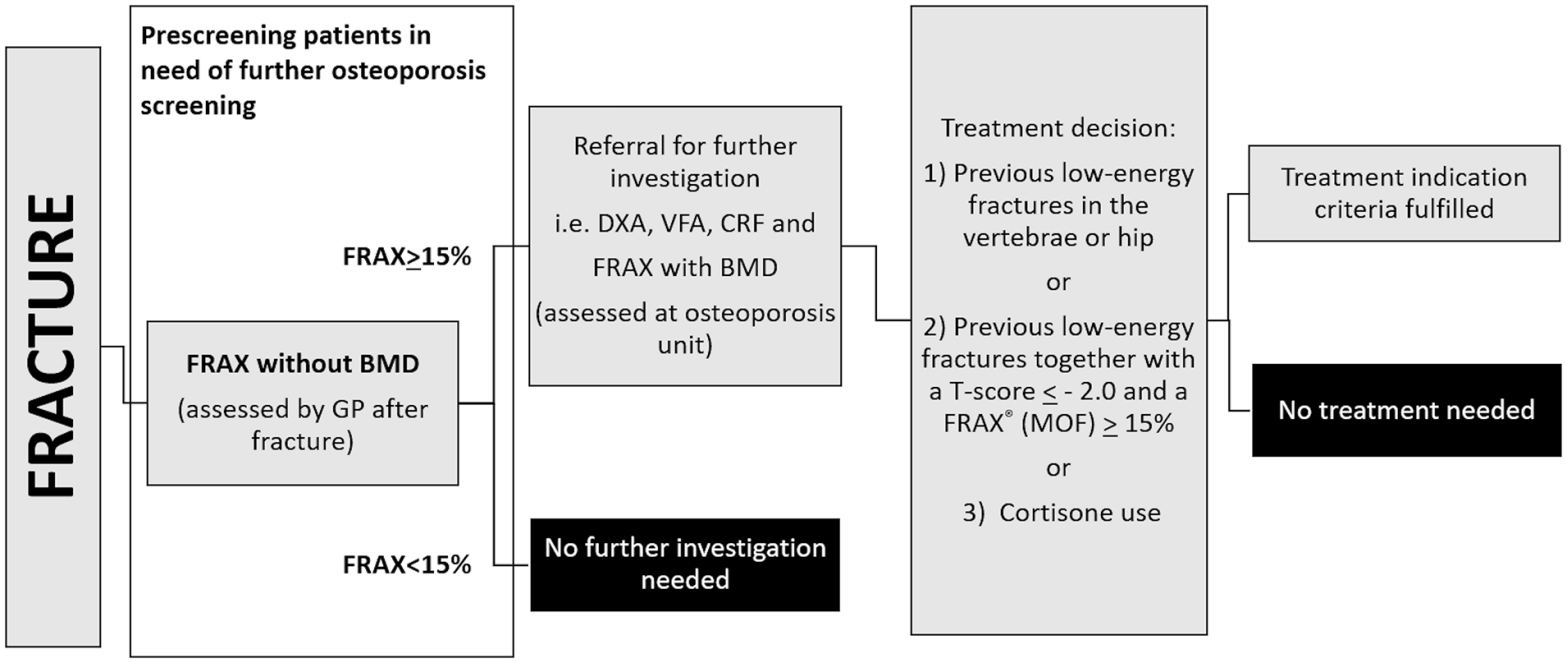
Current FLS for evaluating patients after a low-energy fracture. In this FLS design, FRAX^®^ is an important step to determine which patients need a full osteoporosis evaluation. FRAX^®^ cutoff levels vary between local guidelines. BMD: bone mass density; CRF: clinical risk factor; DXA: dual-energy X-ray absorptiometry; DXR: digital X-ray radiogrammetry; FLS: fracture liaison service; FRAX^®^: fracture risk assessment tool; GP: general practitioner; VFA: vertebral fracture assessment.

There are several risk assessment tools used to select patients for either further osteoporosis screening or treatment decisions, for example, fracture risk assessment tool (FRAX^®^), QFracture^®^, and Garvan Fracture Risk Calculator.^10,11^ FRAX^®^ is a web-based tool that is widely used internationally for calculating the country-specific 10-year risk of major osteoporotic fractures (MOFs) and hip fractures, based on various CRFs.^12,13^ Figure 1 shows a local FLS, which is in line with several other regional FLSs, and which include FRAX^®^ without bone mass density (BMD) as a major prescreening step for determining which patients should undergo a full osteoporosis evaluation and a treatment decision. The effectiveness of FRAX^®^ without BMD as a prescreening tool in a high-risk cohort is not established.

Digital X-ray radiogrammetry (DXR) is an indirect method of estimating BMD using a hand X-ray scan, involving calculations based on an average geometric measure (cortical thickness) and a structural measure (cortical porosity).^14,15^ DXR-BMD has been shown to be associated with fracture risk^14,15^ and has therefore previously been proposed as an osteoporosis screening method for FLSs. However, to the best of our knowledge, it has not been implemented in an FLS. DXR could be fairly easily implemented in acute or semiacute settings.

Studies on the effectiveness of prescreening steps in FLS are scarce. In the present pilot study, we aimed to investigate the effectiveness of: (1) FRAX^®^ without BMD, that is, the currently used prescreening tool in our FLS; and (2) BMD assessment by DXR and DXA, both of them possible prescreening methods which could be arranged in an acute or semiacute setting.

## Methods

### Research design and methods

Women aged >55 years with a low-energy distal radius fracture were invited to participate in this prospective cohort study during a 2-year time period. Since the main project also aimed to investigate fracture healing,^16^ patient inclusion was finalized within 24 h of the fracture taking place. Patients with an obvious indication for fracture surgery at emergency department (ED) admission were excluded because of the protocol for the fracture healing study. The fracture healing part of the project was not included in this substudy, and data are shown elsewhere.^16^ Besides fracture surgery indication, no other exclusion criteria were applied. Thus, comorbidities, medications, etc., were accepted resulting in an unselected postfracture cohort.

**Figure 2.**
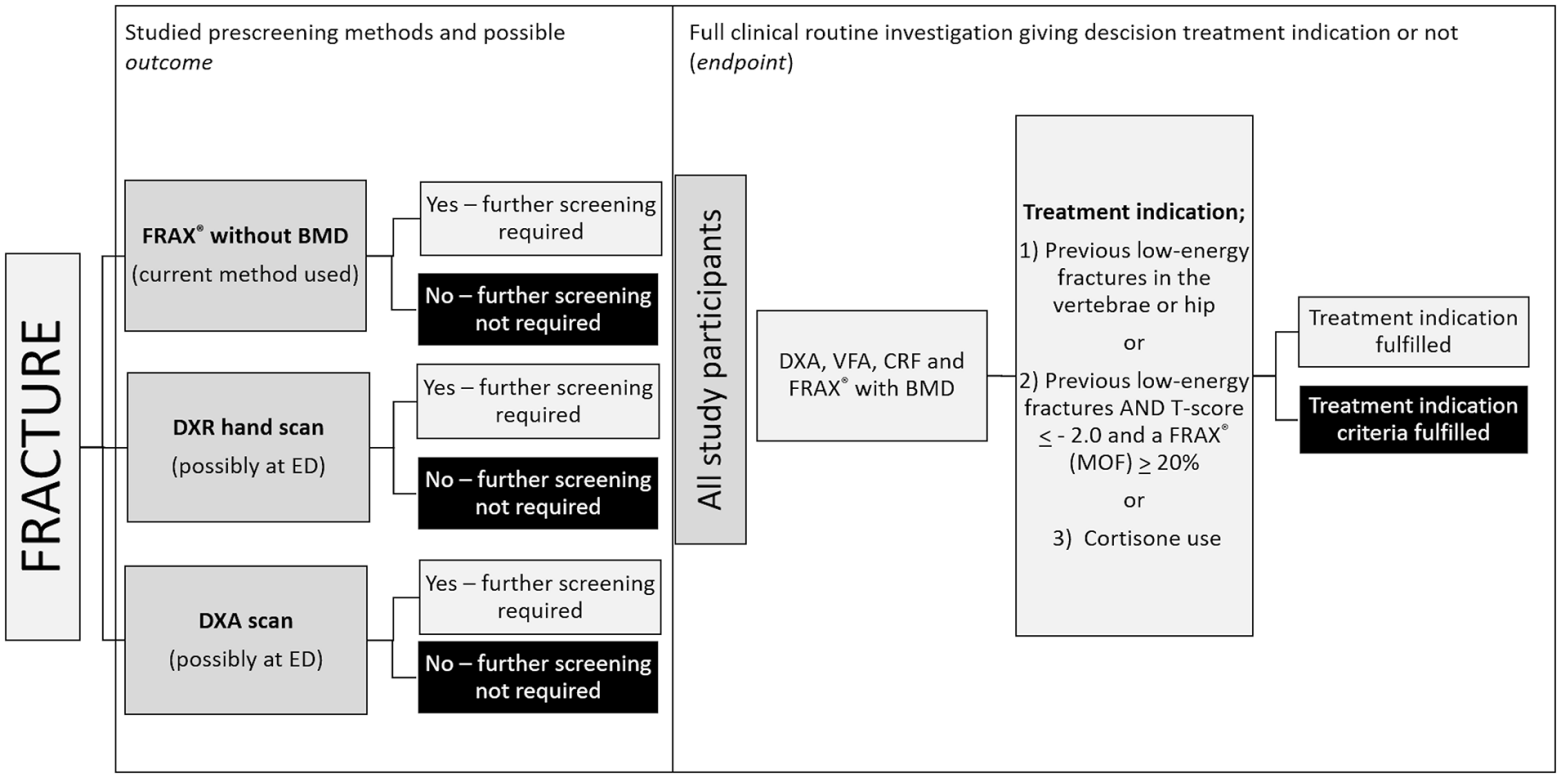
Study design. The precision of the current prescreening method (involving FRAX^®^) was studied and compared to other possible methods, that is, BMD assessment by DXR or DXA in the acute or semiacute setting. BMD: bone mass density; CRF: clinical risk factor; DXA: dual-energy X-ray absorptiometry; DXR: digital X-ray radiogrammetry; ED: Emergency department; FRAX^®^: fracture risk assessment tool; VFA: vertebral fracture assessment.

An overview of the protocol for the current study is shown in Figure 2. All participants underwent: (1) FRAX^®^ without BMD (according to the current local clinical guidelines); (2) DXR (for the purposes of this study); and (3) DXA (for the purposes of this study). In addition, for the purposes of assessing the study endpoint, all participants underwent VFA and CRF assessments. The results of these assessments, together with DXA-BMD, were used to decide whether patients fulfilled the treatment indication criteria or not (endpoint). With an expected sensitivity and specificity of 0.8, prevalence (treatment indication) of 0.5, precision 0.2, and 95% confidence interval of the sample size needed was 39.^17^

### Digital X-ray radiogrammetry

For the purposes of this study, DXR was performed on a conventional unprocessed hand X-ray of the nonfractured arm at the follow-up visit on days 7–10, except for a few patients where, because of misunderstanding, a later scan (within 8 weeks postfracture) was performed. The images were analyzed by Sectra (OneScreen, Sectra AB, Linköping, Sweden), as described in detail by Kälvesten et al.^15^ In brief, measurements were automatically made in the diaphyseal regions of metacarpals II–IV on digital images. Cortical thickness, bone volume, and porosity were determined and used for approximating DXR-BMD.

### Dual-energy X-ray absorptiometry and risk evaluation

BMD was measured using a central DXA system (Hologic Discovery, Hologic, MA, USA). Scans on the hip, spine, and radius were performed by a DXA-certified nurse. The T-score-references (for Caucasian females) supplied by the manufacturer were used. VFAs were carried out on lateral scans to evaluate the potential presence of vertebral fractures and their grades, according to Genant et al.^18^

Data on CRFs were collected from patients using a locally developed clinical risk survey, including FRAX^®^ questions and in-depth interviews by experienced DXA nurses. Data were also obtained from patient records and previous X-rays (plain film and computed tomography (CT) examinations) from the hospital’s picture archiving and communication system (PACS), that is, the X-rays taken at our hospital as part of routine clinical practice.

### Treatment indication

Evaluation of the patients using the treatment indication criteria was conducted by experienced osteoporosis physicians. In line with regional guidelines, the treatment indication criteria were deemed to be fulfilled when patients had one of the following: (1) previous low-energy fractures in the vertebrae or hip (based on information from the patient or a case record); (2) previous low-energy fractures together with a DXA T-score ⩽ −2.0 and a FRAX^®^ with BMD ⩾ 20%; or (3) cortisone use. VFAs were analyzed by three physicians (two osteoporosis physicians and one radiologist). All three had >10 years of clinical experience. All detected vertebral fractures were confirmed on conventional X-rays/CT scans.

### Statistical analysis

IBM SPSS Statistics version 23.0 was used for the statistical analysis. For comparison between the patients who fulfilled the treatment indication criteria and those who did not, the independent *t*-test was used. Furthermore, a receiver operating characteristic (ROC) curve/area under the curve (AUC) analysis was performed to compare the ability of the screening methods to discriminate between patients who did and did not fulfill the treatment indication criteria (“yes” or “no”). Screening method cutoff values indicating the requirement for further patient evaluation were also defined to render a “yes” or “no” outcome. For the DXR and DXA methods, we defined two cutoff levels, that is, T-score ⩽ −2.0 or ⩽−2.5. For FRAX^®^ without BMD, we also defined two cutoff levels, that is, ⩾15% or ⩾20%. In addition, we constructed two combined cutoff levels, that is, “FRAX^®^ without BMD ⩾ 15% and DXR T-score ⩽ −2.5” and “FRAX^®^ without BMD ⩾ 15% and/or DXR T-score ⩽ −2.5.” A *p* value < 0.05 was considered statistically significant.

### Ethics

The study was approved by the Research Ethics Committee of the Faculty of Health Sciences, Linköping University (Dnr 2010/199-31 and Dnr 2011/379-32).

## Results

### Patient characteristics

The patient characteristics are summarized in Table 1. In total, 41 patients (mean age: 70 ± 8 years; range: 60–87 years) were included. Of these, 38 underwent a DXR examination (the remaining three patients had hand X-ray images that, for technical reasons, could not be used for DXR analysis).

**Table 1.** Patient characteristics.

	All (n = 41)	Treatment recommended (n = 22)	Treatment not recommended (n = 19)	*P* value
Risk factor				
Age (years), mean ± SD [range]	70 ± 8 [60–87]	71 ± 8 [60–87]	68 ± 7 [61–85]	*0.29*
Height reduction from peak height (cm), mean ± SD [range]	2.6 ± 2.2 [0–11]	3.0 ± 2.7 [0–11]	2.1 ± 2.71.6 [0–5.5]	*0.23*
FRAX^®^ without BMD (MOF)	27% ± 12%	31% ± 13%	24% ± 9%	*0.051*
Number of patients with FRAX^®^ without BMD (MOF) ⩾ 20%, number (%)	26 (65%)	16 (76%)	10 (53%)	*0.19*
Number of patients with FRAX^®^ without BMD (MOF) ⩾ 15%, number (%)	35 (88%)	19 (91%)	16 (84%)	*0.65*
Number of patients with vertebral fracture on VFA, number (%)	6 (18%)	6 (32%)	0 (0%)	*0.024*
FRAX^®^ with BMD (MOF)	23% ± 11%	28% ± 12%	17% ± 4%	*0.001*
DXR T-score, mean ± SD [median]	−2.0 ± 1.3 [−2.1]	−2.4 ± 1.5 [−2.8]	−1.5 ± 1.0 [−1.4]	*0.03*
DXA T-score (lowest per patient), mean ± SD [median]	−2.3 ± 1.0 [−2.1]	−2.8 ± 1.0 [−2.8]	−1.8 ± 0.7 [−1.9]	<*0.001*

BMD: bone mineral density; DXA: dual-energy X-ray absorptiometry; DXR: digital X-ray radiogrammetry; FRAX^®^: fracture risk assessment tool; MOF: major osteoporotic fracture.

Two patients (5%) were on cortisone medication and one patient was treated with bisphosphonate (>3 months); the latter was excluded from the FRAX^®^ without BMD analysis. Fifteen percent had heredity risk factors for osteoporosis (either parental hip or vertebral fracture, but only the former was used in the FRAX^®^ analysis), and 24% had experienced a low-energy fracture prior to the present one.

FRAX^®^ with BMD was significantly lower, that is, 23% ± 11%, than FRAX^®^ without BMD, that is, 27% ± 12% (*p* = 0.001). Five patients had FRAX^®^ without BMD < 15% (i.e. the limit set in the national guidelines as the cutoff for further osteoporosis evaluation).

In total, 22 patients (54%) fulfilled the treatment indication criteria after being fully evaluated according to routine clinical practice (which included DXA examination, VFA, CRF assessment, and FRAX^®^ with BMD).

### Selection using FRAX^®^ without BMD, DXR, or DXA

The AUC values for selecting patients for further osteoporosis evaluation are shown in Table 2. The highest AUC value (0.73) was seen using either DXA (T-score ⩽ −2.5 or ⩽−2.0) or DXR (T-score ⩽ −2.5). FRAX^®^ without BMD alone had the lowest AUC values (0.50 for FRAX^®^ without BMD ⩾ 15%). Combining FRAX^®^ with DXR results only yielded slightly better AUC values (0.53–0.60).

**Table 2.** Prescreening methods to identify patients who fulfill the treatment indication criteria.

	AUC	Prevalence (%)	Sensitivity (%)	Specificity (%)	Positive predictive value (%)	Negative predictive value (%)	False negative (%)	False positive (%)
FRAX^®^ ⩾ 15%	0.50	53	90	16	54	60	10	84
FRAX^®^ ⩾ 20%	0.59	53	76	47	62	64	24	53
DXR ⩽ −2.0	0.62	53	65	61	65	61	35	39
DXR ⩽ −2.5	0.73	53	60	89	86	67	40	11
DXA ⩽ −2.0	0.73	54	86	63	73	80	14	37
DXA ⩽ −2.5	0.73	54	59	90	87	65	41	10
DXR ⩽ −2 and/or FRAX^®^ ⩾ 15%	0.60	52	100	6	53	100	0	94
DXR ⩽ −2 *and* FRAX^®^ ⩾ 15%	0.53	51	53	67	63	57	47	33

AUC: area under the curve; DXA: dual-energy X-ray absorptiometry; DXR: digital X-ray radiogrammetry; FRAX^®^: fracture risk assessment tool.

The highest sensitivity (Table 2) was seen using FRAX^®^ without BMD ⩾ 15% (90%), followed by DXA ⩽ −2.0 (86%), DXR ⩽ −2.0 (68%), DXR ⩽ −2.5 (60%), and DXA ⩽ −2.5 (59%). Corresponding figures for specificity were 16% (FRAX^®^ without BMD), 63% (DXA ⩽ −2.0), 58% (DXR ⩽ −2.0), 89% (DXR ⩽ −2.5), and 89% (DXA ⩽ −2.5).

Flowcharts illustrating the results of the different prescreening methods are shown in Supplementary Fig. S1A–D. Using FRAX^®^ without BMD ⩾ 15%, that is, our currently used prescreening method, only five patients were determined not to need any further evaluation; however, two of these five patients (40%) actually fulfilled the treatment indication criteria.

## Discussion

In this Swedish study of high-risk individuals, that is, postmenopausal women with a low-energy fracture, we showed that FRAX^®^ without BMD, the method currently used for prescreening in our FLS, had the poorest AUC value (0.5) but a high sensitivity (90%). As potential alternative prescreening steps, BMD assessment by DXR and DXA had better AUC values, but lower sensitivity. Knowledge of the screening tools’ properties (e.g. in terms of AUC values, sensitivity, specificity, and predictive values) is important to compare the tools to identify those with the most acceptable levels of overestimation and underestimation, where overestimation, that is, a high number of false positives, would unnecessarily increase healthcare costs, whereas underestimation, that is, false negatives, would cause patients to miss out on potentially appropriate osteoporosis treatment.

Regarding FRAX^®^ without BMD, the sensitivity was good (90%), but at the expense of poor specificity (16%). Furthermore, the PPV was only 54%; thus, the FRAX^®^ without BMD value indicated that the majority of patients in the study needed further osteoporosis evaluation; however, only half of them actually fulfilled the treatment indication criteria after a full evaluation.

These data indicate that prescreening with FRAX^®^ without BMD will result in a high number of false-positive individuals, that is, over screening with potential health economic consequences and a lower, but still a considerable number of false negatives resulting in missed treatment initiations. These results indicate that the FRAX^®^ method with a cutoff of 15% might not be an optimal method to prescreening patients for further osteoporosis evaluation in this particular setting, that is, high-risk cohort. Similarly, a poor precision of prescreening with FRAX^®^ was found when investigating a cohort of people living with HIV, where the recommended screening cutoff of FRAX^®^ > 10% showed a poor prediction of low BMD, that is, osteoporosis.^19^ FRAX^®^ was developed based on fracture outcome, that is, to predict future fractures and not for detection of low BMD or treatment indication. This might explain the poor outcome in the studies discussed above and also illustrates the need of introducing these kinds of tools, that is, FRAX^®^, cautiously in a prescreening purpose.

Theoretically, our results are not surprising since the average Swedish woman achieves a FRAX^®^ > 15% by the age of 60 years after a low-energy fracture but with no other CRF (mean height: 166 cm and mean weight: 70 kg, for Swedish women aged 50–59 years, according to data from Statistics Sweden^20^ (Statistiska centralbyrån, SCB)). The corresponding age for men is 73 years (mean height: 179 cm and mean weight: 86 kg).

Although FRAX^®^ is easily available and free, other prescreening methods may be more efficient and still suitable in the clinical process for fracture patients. Since many potential osteoporosis patients are lost to follow up after experiencing a low-energy fracture, it would be beneficial to assess the patients’ risk profile in the acute or semiacute settings. We therefore raised the question of whether assessing BMD, by DXR or DXA analysis, at the ED or by postfracture healing X-ray controls, might be efficient. The DXR method is not based on a dual-energy technique as in ordinary DXA scans, but it instead uses geometric and structural measures on monoenergetic plane X-ray images to approximate BMD.^15^ However, the BMD from DXR correlated significantly with the central DXA-BMD values at all scanned sites except for the hip neck (data not shown). To our knowledge, no prior study has focused on DXR as a prescreening method for further osteoporosis evaluation in the postfracture process, though some studies have investigated DXR as a predictor of future fracture risk.^14,15^ In our study, using DXR (cutoff: T-score ⩽ −2.5) as a prescreening method yielded an AUC value of 0.73, which was higher than that for FRAX^®^. DXR had better specificity, PPV, and NPV but lower sensitivity than FRAX^®^. Thus, four of 10 patients who fulfilled the treatment indication criteria were falsely declared healthy (false negative) by the DXR method. In contrast to FRAX^®^, the use of DXR imposes extra costs on the healthcare system, which should be considered when judging the value of potential prescreening methods.

Besides FRAX^®^ and DXR, we also studied DXA as a prescreening method. Currently, a DXA scan together with CRF assessment and VFA is the gold standard for treatment decisions. Furthermore, many FLSs offer all postfracture patients a full evaluation, including DXA, VFA, and CRF assessment, that is, without a prescreening step. However, if we could hypothetically perform a plain DXA analysis (without VFA or CRF assessment) in the acute or semiacute setting, the AUC would be 0.73 (using a T-score of either ⩽−2.5 or −2.0 as the cutoff level). The higher T-score cutoff (−2.0) would yield a better sensitivity than the lower T-score cutoff (⩽−2.5) (86% versus 59%). This sensitivity is similar to that of FRAX^®^ (91%) but shows a better specificity (63% versus 16%).

Undiagnosed vertebral fractures are common^9^ and pose a challenge in osteoporosis care, especially since a vertebral fracture is a strong risk factor for new, potentially avoidable fractures and thus important to act on. This was confirmed in our study, since unknown vertebral fractures were found in a fifth of the patients. To detect these patients, VFA should ideally have been conducted in the acute/semiacute setting. When combining DXA and VFA (without taking into account other CRFs) as a prescreening method, the figures improved further: AUC: 0.78, sensitivity: 96%, specificity: 63%, PPV: 75%, and NPV: 92%.

Our study has some limitations, including that the studied cohort was small, and thus the results need to be confirmed in larger cohort studies. We see the current study as a pilot study. Only women with an age >55 years were included in the study, which makes a generalization of the results unfeasible. Patients with an obvious indication for fracture surgery at ED admission were excluded because of the protocol for the fracture healing study. This might have led to the exclusion of patients with more severe osteoporosis. Treatment indication may differ locally and change over time, which may affect the precision of prescreening tests. Newly published national recommendations (2021), not locally implemented, recommends a higher FRAX cutoff (20%) and higher T-score (−1) for treatment initiation. However, prospectively applying these recommendations on our data set (Supplement 2) yielded similar results regarding FRAX^®^ prescreening (AUC 0.55), but slightly lower regarding DXA and DXR cutoff −1.0 (AUC 0.59 and 0.54, respectively).

## Conclusion

In conclusion, the results of this pilot study raise questions about the effectiveness of using prescreening steps in FLSs for high-risk individuals, that is, postmenopausal Swedish women with low-energy fractures. In our cohort, the FRAX^®^ without BMD method had the lowest precision, that is, both over- and underestimated patients in need of further osteoporosis evaluation. Some FLS designs exclude prescreening steps and recommend that all patients undergo DXA examination, VFA, and CRF assessment in the postfracture setting. The results of our study, though it was small, support this FLS design, especially in this cohort of high-risk patients.

## Supplemental Material

sj-docx-1-smo-10.1177_20503121211073421 – Supplemental material for Selection of risk assessment methods for osteoporosis screening in postmenopausal women with low-energy fractures: A comparison of fracture risk assessment tool, digital X-ray radiogrammetry, and dual-energy X-ray absorptiometry Click here for additional data file. Supplemental material, sj-docx-1-smo-10.1177_20503121211073421 for Selection of risk assessment methods for osteoporosis screening in postmenopausal women with low-energy fractures: A comparison of fracture risk assessment tool, digital X-ray radiogrammetry, and dual-energy X-ray absorptiometry by Mischa Woisetschläger, Simona Chisalita, Marta Vergara and Anna Spångeus in SAGE Open Medicine

sj-tif-2-smo-10.1177_20503121211073421 – Supplemental material for Selection of risk assessment methods for osteoporosis screening in postmenopausal women with low-energy fractures: A comparison of fracture risk assessment tool, digital X-ray radiogrammetry, and dual-energy X-ray absorptiometry Click here for additional data file. Supplemental material, sj-tif-2-smo-10.1177_20503121211073421 for Selection of risk assessment methods for osteoporosis screening in postmenopausal women with low-energy fractures: A comparison of fracture risk assessment tool, digital X-ray radiogrammetry, and dual-energy X-ray absorptiometry by Mischa Woisetschläger, Simona Chisalita, Marta Vergara and Anna Spångeus in SAGE Open Medicine
