# Supplementary material for: Selection of risk assessment methods for osteoporosis screening in postmenopausal women with low-energy fractures: A comparison of fracture risk assessment tool, digital X-ray radiogrammetry, and dual-energy X-ray absorptiometry
Source: SAGE Open Med. 2022 Jan 15;10:20503121211073421. doi: 10.1177/20503121211073421 (PMC8771752; doi:10.1177/20503121211073421)
Supplement: sj-docx-1-smo-10.1177_20503121211073421 – Supplemental material for Selection of risk assessment methods for osteoporosis screening in postmenopausal women with low-energy fractures: A comparison of fracture risk assessment tool, digital X-ray radiogrammetry, and dual-energy X-ray absorptiometry [file sj-docx-1-smo-10.1177_20503121211073421.docx]

Supplement 2. Alternative treatment indication (new national recommendations 2021)

|  | **AUC** | **Prevalence** | **Sensitivity** | **Specificity** | **Positive predictive value** | **Negative predictive value** |
| --- | --- | --- | --- | --- | --- | --- |
| FRAX >15 | 0.55 | 58% | 91% | 18% | 60% | 60% |
| DXR < -1.0 | 0.54 | 58% | 77% | 31% | 61% | 50% |
| DXA < -1.0 | 0.59 | 59% | 100% | 18% | 63% | 100% |

AUC = area under the curve, FRAX® = Fracture Risk Assessment Tool, DXR=digital X-ray radiogrammetry, DXA = dual-energy X-ray absorptiometry
