# Supplementary figures and images for: Selection of risk assessment methods for osteoporosis screening in postmenopausal women with low-energy fractures: A comparison of fracture risk assessment tool, digital X-ray radiogrammetry, and dual-energy X-ray absorptiometry
Source: SAGE Open Med. 2022 Jan 15;10:20503121211073421. doi: 10.1177/20503121211073421 (PMC8771752; doi:10.1177/20503121211073421)

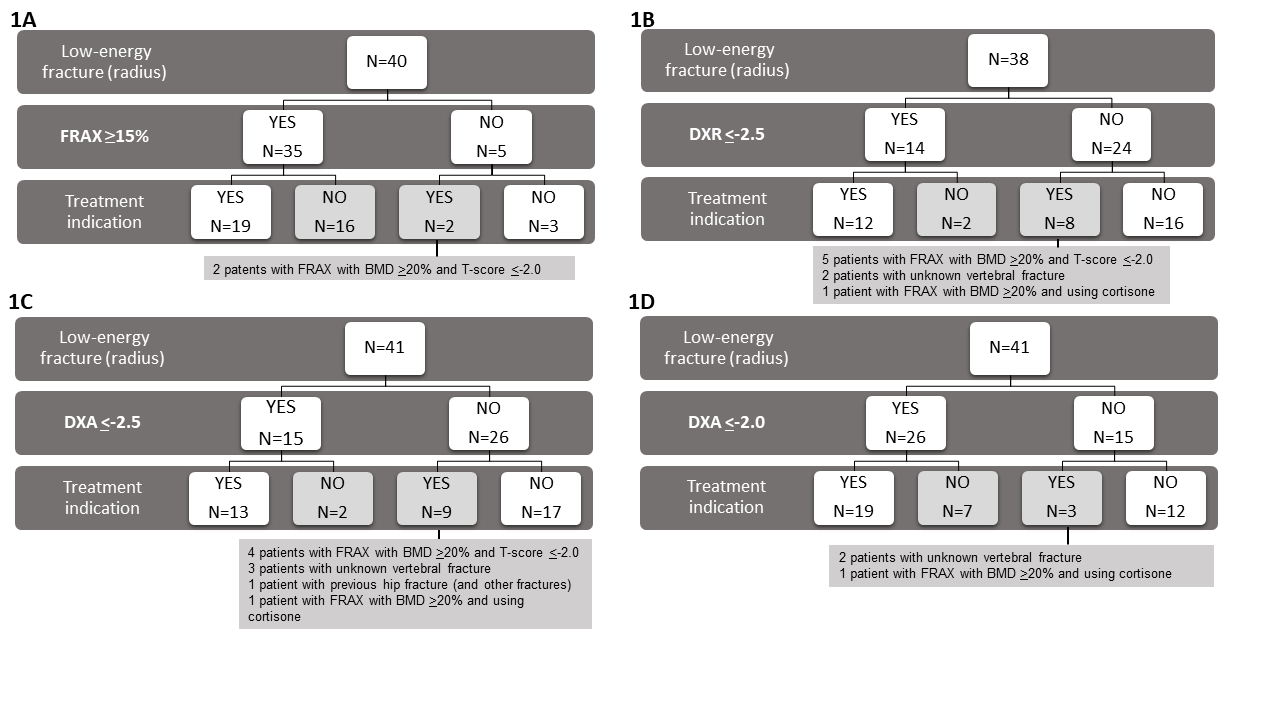

Supplement: sj-tif-2-smo-10.1177_20503121211073421 – Supplemental material for Selection of risk assessment methods for osteoporosis screening in postmenopausal women with low-energy fractures: A comparison of fracture risk assessment tool, digital X-ray radiogrammetry, and dual-energy X-ray absorptiometry [file sj-tif-2-smo-10.1177_20503121211073421.tif]
